# Effect of antibody switch in non-responders to a CGRP receptor antibody treatment in migraine: A multi-center retrospective cohort study

**DOI:** 10.1177/03331024211048765

**Published:** 2021-10-13

**Authors:** Lucas Hendrik Overeem, Andreas Peikert, Maxi Dana Hofacker, Katharina Kamm, Ruth Ruscheweyh, Astrid Gendolla, Bianca Raffaelli, Uwe Reuter, Lars Neeb

**Affiliations:** 1 Department of Neurology, Charité – Universitätsmedizin Berlin, Berlin, Germany; 2 Neurologicum Bremen, Bremen, Germany; 3 Department of Neurology, Ludwig Maximilians University Munich, Munich, Germany; 4 Praxis Gendolla, Essen, Germany; 5 Universitätsmedizin Greifswald, Greifswald, Germany

**Keywords:** Migraine, switch, responder rate, erenumab, galcanezumab, fremanezumab

## Abstract

**Background:**

Switching between antibody classes might be a treatment option in migraine patients who have not responded to one class of a CGRP-(receptor) monoclonal antibody (mAb), but there are no efficacy data so far. In this real-world analysis, we assessed the treatment response to a CGRP-mAb in patients that have previously failed the CGRP-receptor-mAb erenumab.

**Methods:**

We analyzed retrospective headache diary data of 78 patients with migraine who switched between CGRP-mAbs classes at four German headache centers either due to lack of efficacy or intolerable side effects. Among these, we identified 25 patients who did not respond to erenumab after three treatment cycles (defined as <30% reduction of monthly headache days) and had complete headache documentation at least one month before and during both treatments. We assessed the ≥30% responder rate at month three after switching from erenumab to a CGRP-mAb (galcanezumab or fremanezumab) (primary endpoint). Secondary endpoints included ≥50% responder rate, monthly headache days, and monthly days with acute medication use. In an exploratory subgroup analysis patients were stratified for daily and non-daily headache.

**Results:**

The switch from erenumab to a CGRP-mAb led to a ≥30% response in one-third (32%) of the patients after three treatment cycles. A ≥50% response was achieved in 12% of the patients. Monthly headache days were reduced in month three compared to baseline (20.8 ± 7.1 to 17.8 ± 9.1; p = 0.009). Stratified analysis revealed that no patient with daily headache (n = 9) responded to the treatment switch, while a 30% response was achieved by 50% of patients with non-daily headache (n = 16).

**Conclusion:**

Our findings demonstrate that a relevant proportion of erenumab non-responders might benefit from a treatment switch to a CGRP-mAb. Switching seems to be a promising treatment option especially in migraine patients with non-daily headache.

## Introduction

The neuropeptide calcitonin gene-related peptide (CGRP) is a potent endogenous vasodilator and a key neurotransmitter in the pathophysiology of migraine (1,2). Two classes of monoclonal antibodies (mAb) targeting CGRP or its receptor have been proven effective and safe in the prevention of episodic (EM) and chronic migraine (CM) (3). To date, two CGRP monoclonal antibodies (CGRP-mAb), fremanezumab and galcanezumab, and one CGRP receptor monoclonal antibody (CGRP-R-mAb), erenumab, have been approved for migraine prevention in Europe.

About 15–25% of the patients treated with a CGRP-(receptor)-mAb (CGRP-(R)-mAb) discontinue treatment due to lack of efficacy (4,5). In these patients switching of CGRP-mAb classes may be an option. Because of the different targets of the two CGRP-mAb classes (ligand vs. receptor), it is conceivable that patients who did not respond to one CGRP-mAb class may benefit from a switch to the other class. Clinical observations support this hypothesis: one small case series reported three patients who did not respond to treatment with erenumab showed a substantial reduction of headache days after a switch to galcanezumab (6). Other than this case report no clinical trial assessed the effect of the switch of non-responders from one CGRP-mAb class to the subsequent exposure of another CGRP-mAb. In general, the effects of the common practice of switching between different classes of oral migraine preventive medication have not been investigated thoroughly. One study shows that adherence to oral preventive medications worsens as patients cycle through the various oral treatment options (7). 

In this retrospective multi-center cohort study, we aimed to evaluate the therapeutic benefit in migraine patients who switched from prophylactic treatment with a CGRP-R-mAb to a CGRP-mAb due to non-response. To do so, we retrospectively reviewed headache diaries and clinical data of these patients at four German headache centers.

## Methods

### Study design

This retrospective longitudinal cohort study was performed at four German headache centers/private neurological practices specialized in headache treatment (Charité – Universitätsmedizin Berlin, Ludwig Maximilian University Munich, Neurologicum in Bremen, and Praxis Gendolla in Essen)

**Figure 1.**
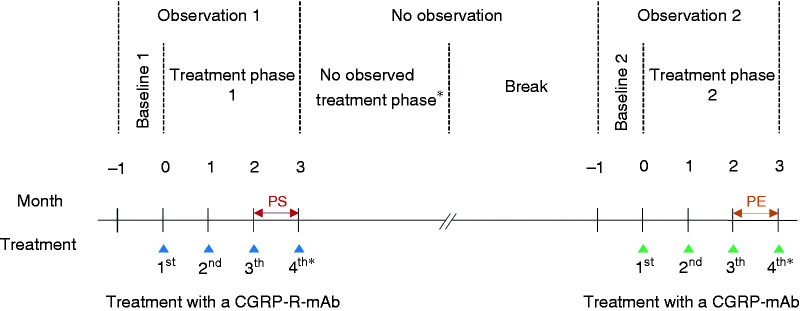
Timeline of study. PS = patient selection, based on the Δ from this period compared to baseline 1, patients were selected in our cohort; PE = primary endpoint, the primary endpoint was based on the Δ from this period compared to baseline 1. * Some patients continued treatment after week 12 (not observed)

The study consisted of two observational periods of 16 weeks. Each observational period was divided into a baseline of four weeks (pre-treatment) and a treatment phase of three months (three injection cycles) with a varying break in between (no observation). During the first treatment phase, patients were treated with the CGRP-R-mAb erenumab, and during the second treatment phase with one of the CGRP-mAbs (galcanezumab or fremanezumab). Each observational period consisted of four epochs: week −4 to −1 (baseline) and during treatment week 1 to 4 (month 1), week 5 to 8 (month 2), and week 9 to 12 (month 3) (Figure 1). For this study, a month was defined as 28 days.

Because erenumab was approved earlier than galcanezumab and fremanezumab in Europe, we focused on the switch from the CGRP-R-mAb erenumab to a CGRP-mAb in this analysis.

We conducted this study according to the declaration of Helsinki. The local ethics committee approved the study (EA1/154/20). Written informed consent for participation was not required for this retrospective study of data acquired during routine medical treatment by the national legislation and the institutional requirements. Our report complies with the “Strengthening the Reporting of Observational Studies in Epidemiology” (STROBE) Statement for cohort studies.

### Patient selection

Participating centers screened all migraine patients who received a CGRP-R-mAb (erenumab) or a CGRP-mAb (galcanezumab or fremanezumab) between November 2018 and May 2020. To avoid selection bias, study sites shared their data of all eligible patients 18 years or older, who had a diagnosis of episodic or chronic migraine with or without aura according to the International Classification of Headache Disorders, 3rd edition (ICHD-3) (8), who had received at least two CGRP-(R)-mAbs and had a headache diary in file with minimum documentation of the last treatment month with the first CGRP antibody and first two months of the second antibody. Data from patients who had previously participated in a CGRP-mAb clinical trial was not shared. Data was shared independent of treatment effects or cause for the antibody switch (lack of efficacy, partial efficacy, or side effects).

Patients were included in the final analysis if they (i) received at least three injections of erenumab followed by at least two injections of a CGRP-mAb, (ii) had complete headache documentation, and (iii) did not discontinue treatment with erenumab because of side effects. Complete headache documentation was defined as headache documentation of at least one month before the first injection (baseline) and three months during the first treatment phase and two months during the second treatment phase.

Patients were excluded if (i) they had a ≥30% reduction of monthly headache days (MHDs) during the third treatment cycle with erenumab. Therewith our study population only consists of patients who were non-responders to erenumab (defined as a <30% reduction of MHDs at month 3 of treatment).

### Variables, data extraction, endpoints, and missing data

We extracted the number of MHDs and the days with acute medication use (AMD) from the headache diaries. A headache day was defined as any day on which a patient recorded any type of headache. Because of the non-standardized headache diaries and the varying details of documentation of headache characteristics and accompanying symptoms during each headache attack, reliable differentiation between headache and migraine days was not possible. Therefore, responder analysis according to MHDs was used as the primary endpoint. In the case of missing headache documentation from month 3, during the second treatment phase, either due to discontinued treatment or missing diary data, we assumed no further change and used headache data from month 2 for analyses (last observation carried forward approach).

All endpoint analyses compared baseline data in the second observation period with the findings in month 3 (after the CGRP-mAb switch). The primary endpoint was the ≥30% reduction in MHDs from baseline in month 3 (≥30% responder rate). Secondary endpoints were the ≥50% reduction in MHDs from baseline in month 3 (≥50% responder rate) and the change from baseline in MHDs and AMDs in month 3 after the switch of CGRP-mAb class.

Moreover, we extracted the patient characteristics age, gender, migraine diagnoses, migraine years as well as prior prophylactic treatments, concomitant prophylactic treatment, and type and dose of CGRP-(R)-mAb from the patient records. Continuous variables are expressed in mean (standard deviation), and categorical variables in n (%).

### Statistical Analyses

Normal distribution of data was assessed with the Kolmogorov–Smirnov test. Since data of MHDs and AMDs were not normally distributed we used non-parametric tests for analysis. We used Friedman’s 2-way ANOVA by ranks test (for k samples) for repeated measurements with all-time points as factor (baseline, month 1, month 2, and month 3). In case of a significant main effect, a Dunn’s pairwise posthoc test was carried out and a Bonferroni correction for multiple testing was applied. Due to the robustness to outliers in non-parametric testing, we did not take outliers into account. A value of p ≤ 0.05 was considered statistically significant. Statistical analyses were performed with IBM SPSS Statistics, version 25 (IBM, Armonk, NY, USA).

To better understand the effect of a switch for certain subpopulations, we stratified for the presence of daily headache (defined as headache on 28/28 days during baseline 1). Because of the retrospective design of the study, we did not perform a sample size calculation. The study size was achieved depending on the number of cases fulfilling the inclusion criteria treated at the participating centers and the completeness of the headache documentation.

## Results

**Figure 2.**
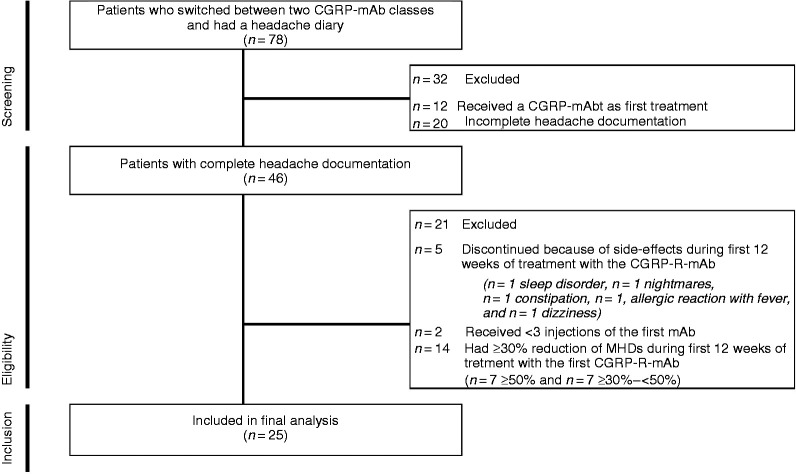
Flow chart of patient selection for final analysis.

At all centers, 1383 patients received a CGRP-(R)-mAb between November 2018 and May 2020. We identified 78 patients who switched between two CGRP-mAb classes and had available documentation of headache diaries (n = 29 Berlin; n = 28 Bremen; n = 8 Essen; n = 13 Munich). Patient disposition and reasons for exclusion are displayed in the flow chart (Figure 2). The final analysis includes data from 25 patients. In four cases with ongoing treatment, headache data for month 3 was not available during the second treatment phase.

### Demography

Of the 25 patients included, 17 (68%) were female. The mean age was 46.5 ± 10.1 years at baseline of the first treatment phase. The mean duration of migraine was 25.7 ± 10.3 years. The majority of patients (n = 22; 88%) were diagnosed with chronic migraine. A history of aura was reported in 12 patients (48%) patients. Per inclusion criteria, all patients received erenumab as the first CGRP-mAb therapy. In the second treatment phase, 12 (48%) patients received galcanezumab, and 13 (52%) patients received fremanezumab. Our cohort included 16 patients with non-daily headache (<28 out of 28 days) and nine patients with daily headache (28 out of 28 days). In the latter group, there was no history of preceding chronic tension-type headache nor comorbidity with new daily persisting headache or any symptomatic headaches apart of medication overuse headache. (Table 1).

**Table 1.** Baseline characteristics and demographics.

		Stratified	
	Total cohort	Non-daily headache	Dailyheadache
	n = 25	n = 16	n = 9
Characteristics
Age in years, mean (SD)	46.5 (10.1)	47.4 (11.5)	44.8 (7.2)
Female, n (%)	17 (68)	12 (75)	5 (56)
Chronic migraine, n (%)	22 (88)	14 (88)	9 (100)
Aura, n (%)	12 (48)	6 (38)	6 (67)
Migraine years, mean (SD)	25.7 (10.3)	29.9 (9.3)	19.2 (8.5)
First monoclonal antibody			
Erenumab, n (%)	25 (100)	16 (100)	9 (100)
Started with initial dose of 70 mg, n (%)	25 (100)	16 (100)	9 (100)
Increased dose to 140 mg, n (%)	23 (92)	15 (94)	8 (89)
Baseline headache days, mean (SD)	21.1 (6.7)	17.2 (5.3)	28 (0.0)
Baseline acute medication days, mean (SD)	8.5 (3.0)	8.8 (3.0)	8.1 (3.2)
№ of patients with MO at baseline, n (%)^a^	6 (24)	4 (25)	2 (22)
Concomitant prophylaxis, n (%)	7 (28)	2 (13)	5 (56)
Break			
Mean duration break in days, mean (SD)^b^	93.4 (55.9)	87.4 (44.1)	104.1 (74.3)
Concomitant prophylaxis, n (%)	6 (24)	2 (13)	4 (44)
Second monoclonal antibody			
Galcanezumab, n (%)	12 (48)	7 (44)	5 (56)
Fremanezumab, n (%)	13 (52)	9 (56)	4 (44)
Baseline headache days, mean (SD)	20.8 (7.1)	17.1 (6.1)	27.6 (1.3)
Baseline acute medication days, mean (SD)	9.1 (4.7)	9.2 (4.6)	9.0 (5.3)
№ of patients with MO at baseline, n (%)^a^	7 (28)	4 (25)	3 (33)
Concomitant prophylaxis, n (%)	6 (24)	2 (13)	4 (44)

SD = standard deviation; mAb = monoclonal antibody; MO = medication overuse.

^a^MO defined as: intake of any combination of ergotamine, triptans, non-opioid analgesics and/or opioids on a total of ≥10 days/month.

^b^Duration in days between the last injection of the first monoclonal antibody and the first injection of the second monoclonal antibody.

### Prior and concomitant prophylactic therapy

Before erenumab therapy patients had on average 5.6 ± 1.8 non-successful prior first or second-line migraine prophylactics according to the German guideline for migraine prevention (9). More than 95% of patients had previously received botulinum toxin A, topiramate, and amitriptyline. Reasons for discontinuation were lack of efficacy (71%), side effects (16%), both lack of efficacy and side effects (7%), and unclear/unknown reason (6%). Details of prior prophylactic treatments are shown in Table 2.

**Table 2.** Overview of prior prophylactic treatments (n = 25).

	Treated with preventative	Contraindicated preventative
Total of prior prophylactic treatment, mean (SD)^a^	5.6 (1.8)	0.6 (1.0)
Anticonvulsants		
Topiramate, n (%)	25 (100)	–
Valproic acid, n (%)	7 (28)	3 (12)
Toxins		
Botulinum toxin A, n (%)	25 (100)	–
Antidepressants		
Amitriptyline, n (%)	24 (96)	–
Venlafaxine, n (%)	7 (28)	–
Nortriptyline, n (%)	1 (4)	–
Beta-blockers		
Metoprolol, n (%)	23 (92)	2 (8)
Propranolol, n (%)	5 (20)	2 (8)
Bisoprolol, n (%)	2 (8)	–
Calcium antagonist		
Flunarizine, n (%)	14 (56)	9 (36)
Angiotensin receptor blocker
Candesartan, n (%)	3 (12)	–

SD = standard deviation

Seven patients received a concomitant prophylactic treatment with a potential therapeutic effect during the first and/or second observation period. From six of those patients, the concomitant prophylactic treatment remained stable during both periods without any change of drug or dose (n = 1 amitriptyline 50 mg/d; n = 1 topiramate 100 mg/d and venlafaxine 75 mg/d; n = 1 topiramate 100 mg/d, venlafaxine 75 mg/d, and candesartan 8 mg/d (prescribed with another indication than migraine (hypertension)); n = 1 metoprolol 25 mg/d and amitriptyline 125 mg/d; n = 1 amitriptyline 25 mg/d; n = 1 metoprolol 50 mg/d). One patient received a botulinum toxin A treatment (195 Units) during the baseline of observation period 1 which was not continued after the start of erenumab (Table 1).

### Observation period 1

All patients started with 70 mg erenumab and the majority (92%) increased the dose during the first three months to 140 mg. Headache days did not change significantly under erenumab (21.1 ± 6.7 MHDs during baseline and 20.7 ± 6.4 MHDs in month 3). AMDs also remained without significant change in the first treatment phase (8.5 ± 3.0 during baseline and 7.2 ± 3.6 in month 3) (Table 3). Some patients received erenumab for more than three months. Looking at the last month of treatment with erenumab in all patients, no significant change of MHDs and AMDs compared to baseline was observed (MHDs 1.8 ± 4.4; p = 0.637 and AMDs −0.1 ± 5.1; p = 796).

**Table 3.** Monthly headache days and acute medication days during observation period 1.

Observation Period 1			(Change from baseline)		
		Baseline	Month 1	Month 2	Month 3
		Mean	Mean difference	Mean difference	Mean difference
Total cohort	n	(SD)	(95% CI)	(95% CI)	(95% CI)
Monthly Headache Days	25	21.1 (6.7)	−0.6 (−1.8 to 0.6)	−0.6 (−2.0 to 0.8)	−0.4 (−1.6 to 0.9)
Acute Medication Days	17	8.5 (3.0)	−1.4 (−3.9 to 1.0)	−1.5 (−3.5 to 0.5)	−1.4 (−3.0 to 0.3)
Non-daily headache					
Monthly Headache Days	16	17.2 (5.3)	−0.3 (−1.9 to 1.4)	−0.5 (−2.8 to 1.8)	−0.4 (−2.4 to 1.6)
Acute Medication Days	10	8.8 (3.0)	−0.5 (−3.3 to 2.3)	−2.0 (−4.6 to 0.6)	−1.6 (−3.6 to 0.4)
Daily headache					
Monthly Headache Days	9	28.0 (0.0)	−1.3 (−3.1 to 0.4)	−0.8 (−1.6 to 0.1)	−0.3 (−0.9 to 0.2)
Acute Medication Days	7	8.1 (9.0)	−2.7 (−8.1 to 2.7)	−0.7 (−4.8 to 3.4)	−1.0 (−4.8 to 2.8)

SD = standard deviation; 95% CI = 95% confidence interval.

### Results of observation period 2 after switching to a CGRP-mAb

**Figure 3.**
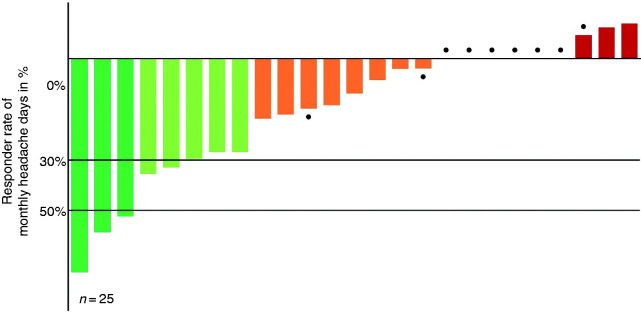
Primary endpoint, individual monthly headache responder rates at month 3 after switch. ● Represents a patient suffering from daily headache.

Patients started with the CGRP-mAb therapy on average 87.4 ± 44.1 days after the last injection of erenumab. Half of the erenumab non-responders received fremanezumab (n = 13; 52%) and half galcanezumab (n = 12; 48%). Five patients discontinued CGRP-mAb treatment after two treatment cycles (four due to lack of efficacy, one due to side effects). In month 3 of CGRP-mAb treatment, eight of 25 patients (32%) archived a ≥ 30% reduction in headache days from baseline 2, and three of these patients (12%) were archived a ≥ 50% reduction in headache days from baseline 2. (Figure 3).

There was a sustained reduction in the mean number of MHDs across the second observation period (p = 0.001). From baseline 2, the MHDs were reduced from 20.8 ± 7.1 days to 18.0 ± 9.0 days in month 2 and 17.8 ± 9.1 days in month 3. The Bonferroni adjusted p-values for multiple testing were respectively p = 0.016 and p = 0.009. No meaningful change in the mean number of AMDs across the second observation period was observed (p = 0.37) (Table 4).

**Table 4.** Monthly headache days and acute medication days after switch during observation period 2.

Observation Period 2			(Change from baseline)			
		Baseline	Month 1	Month 2	Month 3	p-value for repeated measures^a^
		Mean	Mean difference	Mean difference	Mean difference	
Total cohort	n	(SD)	(95% CI)	(95% CI)	(95% CI)	
30% responder rate, n (%)^b^	25		4 (16)	7 (28)	8 (32)	
50% responder rate, n (%)^b^	25		2 (8)	3 (12)	3 (12)	
Monthly Headache Days	25	20.8 (7.1)	−1.5 (−3.1 to 0.2)	−2.9 (−4.8 to −0.9)	−3.1 (−5.0 to −1.2)	0.001
p vs baseline^c^			0.208	0.005	0.003	
Corrected p vs baseline^d^			0.623	0.016	0.009	
Acute Medication Days	17	9.1 (4.7)	−1.6 (−4.1 to 0.8)	−1.7 (−4.1 to 0.6)	−1.9 (−4.3 to 0.4)	0.367
Non-daily headache						
30% responder rate, n (%)^b^	16		4 (25)	7 (44)	8 (50)	
50% responder rate, n (%)^b^	16		2 (13)	3 (19)	3 (19)	
Monthly Headache Days	16	17.1 (6.1)	−1.9 (−4.4 to 0.6)	−4.2 (−6.9 to -1.4)	−4.6 (−7.2 to −1.9)	0.002
p vs baseline^c^			0.171	0.003	0.001	
Corrected p vs baseline^d^			0.513	0.010	0.002	
Acute Medication Days	10	9.2 (4.6)	−1.1 (−4.7 to 2.5)	−1.8 (−4.3 to 0.7)	−2.0 (−5.4 to 1.4)	0.324
Daily headache						
30% responder rate, n (%)^b^	9		0 (0)	0 (0)	0 (0)	
50% responder rate, n (%)^b^	9		0 (0)	0 (0)	0 (0)	
Monthly Headache Days	9	27.6 (1.3)	−0.7 (−2.5 to 1.2)	−0.6 (−2.6 to 1.4)	−0.4 (−1.9 to 1.0)	0.719
Acute Medication Days	7	3.2 (5.3)	−2.4 (−6.6 to 1.8)	−1.6 (−7.3 to 4.2)	−1.9 (−6.3 to 2.6)	0.882

SD = standard deviation; 95% CI = 95% confidence interval.

^a^ Statistical results of repeated measures of the Friedman test for repeated measures.

^b^ Responder rate for monthly headache days.

^c^ p-value for Dunn’s pairwise post-hoc test.

^d^ p-value after Bonferroni correction for multiple testing.

### Stratified analyses for daily and non-daily headache

Stratification for daily headache (DH) revealed that all responders were from the non-daily headache (NDH) group. Subgroup analysis restricted to patients with NDH showed a ≥30% response in 50% and a ≥50% response in 19% of the patients (at month 3). Analysis for the strata DH and NDH showed that only patients with NDH had a substantial decrease of MHDs across the second observation period after the switch (p = 0.002 for NDH; p = 0.72 for DH). No significant changes in AMDs were observed in both strata (Table 4).

## Discussion

This real-world analysis indicates that migraine patients with a non-response to a CGRP-R-mAb treatment may benefit from treatment with a CGRP-mAb. About 32% of the erenumab non-responders showed a clinically meaningful reduction of headache days of more than 30% after switching to a CGRP-mAb. However, patients with daily headache did not benefit from the change of therapy.

To the best of our knowledge, this is the first analysis that assessed the efficacy of CGRP-mAbs in non-responders to previous treatment with erenumab. Patients who stopped erenumab because of partial response or adverse events were not included. The present study investigated the switch from erenumab to a CGRP-mAb because this constellation was most frequently encountered, due to the approval of erenumab preceding that of the CGRP-mAbs in Europe. This data does not allow any comparison of the efficacy of the two different classes of CGRP antibodies and does not indicate that one CGRP-mAb class is superior to the other.

Due to reimbursement restrictions by the German regulatory bodies, patients on CGRP-mAbs had to have no improvement to least 4 (EM) or 5 (CM) first-line migraine preventive medications either due to insufficient response, discontinuation due to side effects, or contraindications (9). These drugs included beta-blockers (metoprolol or propranolol), tricyclic antidepressants (amitriptyline), flunarizine, topiramate, and for CM additionally Onabotulinumtoxin A (BoNT-A). Therefore, this study population belongs to the category of treatment-resistant migraine patients in line with the European Headache Federation (EHF) guidelines (10).

We chose a <30% responder rate as inclusion criteria and a ≥30% responder rate as our primary endpoint. A 30% responder rate is considered clinically meaningful in chronic migraine (11–13) and the vast majority of the patients in this analysis suffered from chronic migraine.

To date, it is not understood why some patients respond to one class of preventives and some do not. This not only applies to the CGRP-mAbs but also to all previously established migraine preventives such as beta-blockers or topiramate. No clinical variables or biomarkers have been identified that allow predicting treatment response. One possible explanation for non-response to CGRP-mAbs might be the existence of different subtypes of migraine, in which CGRP plays a key role in some patients while other neuropeptides such as pituitary adenylate cyclase-activating polypeptide or other mechanisms may play a predominant role in other patients (14). Cernuda-Morollon and co-workers demonstrated that elevated interictal CGRP plasma levels declined in chronic migraine patients that responded to treatment with BoNT-A. High CGRP serum levels seemed to be associated with good treatment response (15,16). To date, no other studies identified biomarkers as possible predictors of treatment response but in the light of specific treatment options with CGRP-(R)-mAbs this will be one important field of upcoming research.

Yet, the hypothesis of neuropeptides with varying importance in migraine subtypes does not apply to the varying response to the different CGRP-(R)-mAb classes demonstrated in our cohort as both classes act on CGRP mediated pathways. The main difference between these two classes is that one mAb blocks CGRP signaling by binding to the CGRP receptor and the other by targeting the ligand directly. This might confer differential effects because erenumab blocks only the CGRP receptor, while CGRP-mAbs block CGRP signaling not only at the CGRP receptor but also at the amylin 1 (AMY_1_) receptor. Currently, it is not known if amylin contributes to the mechanism of migraine and if suppressing CGRP mediated mechanisms on the AMY_1_ receptor has any impact (17,18). However, this might be one possible explanation for the observed effect of the mAb switch. Alternatively, differences between drugs targeting the same pathways may confer differential effectiveness to these drugs, such as is seen with the different triptans.

Three randomized placebo-controlled clinical trials (RCTs) assessed the efficacy of CGRP-(R)-mAbs in reducing monthly migraine days in patients that failed 2–4 previous preventives (19–21). A ≥50% response was archived by 30% of the patients in the LIBERTY study during weeks 9 to 12 (21) and in the CONQUER (20) and FOCUS (19) studies in 37.7% and 34% of the patients during the first 12 weeks of treatment.

Other real-world studies have assessed responder rates and MHDs in patients receiving erenumab (but no previous treatment with another CGRP-mAb). These studies reported a >30% reduction of MHDs in 50–70% of their patients after three months of treatment (22–26).

The ≥50% response rate of MHDs after the mAb switch in our cohort was lower compared to RCTs. This also applies to the 30% response rates of MHDs compared to other real-world studies. It is striking that patients with daily headaches showed no treatment effect in our study. Looking at the sub-group of migraine patients with non-daily headache a ≥30% response was observed in 50% of the patients. A ≥50% response was observed in 19% of the patients. This is in line with previous real-world findings of our group which show a >30% reduction of MHDs in 51% of the patients in a comparable treatment-resistant population of chronic migraine who failed all first-line preventives including BoNT-A but no previous CGRP-R-mAb (22).

Seven patients received a concomitant prophylactic therapy during the treatment with a CGRP-(R)-mAb. All, but two dose regimens (metoprolol 25 mg, amitriptyline 25 mg) were in line with the German treatment guidelines for migraine prophylaxis treatment (9). We do not expect our results to be affected by these medications because concomitant treatments were stable during each treatment phase for all but one patient. One patient received the last treatment with BoNT-A in the month before the start of treatment with erenumab. It is unlikely that this treatment affected our results since the reduction of MHDs was <30% during this period.

Medication overuse (MO) was present in 24% of our study population, which is lower than in most clinical trials in CM patients. MO may influence the results of this real-world analysis due to the small sample size and different types of acute medications leading to MO. However, more recent data from Phase 3 trials indicate that in parallel to the reduction of MMD CGRP-(R)-mAb therapy also leads to a reduction in acute medication intake (27,28).

Patients with daily headache are often considered resistant to preventive treatment. To the best of our knowledge, the treatment response in patients with daily headache has not been studied yet. Based on our observation of the strict non-response in this subgroup future research may focus on treatment response in patients with daily or near-daily headache.

Our study has several strengths and limitations. It is the first cohort study observing the effect of a CGRP-mAb switch in non-responders to previous treatment with a CGRP-R-mAb. The strict exclusion of patients that switched CGRP-mAb classes due to side effects or partial effects allows concentration on the efficacy of the switch in CGRP-R-mAb non-responders. By stratification for patients with and without daily headache, we were able to identify better outcomes in patients with non-daily headache which might be helpful in clinical practice. The main limitation of the study is the retrospective study design and the small number of patients included in the analysis. Due to the retrospective design, routine clinical care headache diaries were used with varying quality of documentation and a significant proportion of patients had to be excluded due to incomplete headache documentation. The observed three-month treatment phase might not have been long enough to exclude patients who might respond after an extended treatment period. Most of our patients had chronic migraine. Placebo-controlled clinical trials of erenumab in chronic migraine patients had a treatment period of three months and no data is available on how many of these patients might respond later. A real-world study observed a small group of patients with chronic migraine who did not respond after three months but achieved a 30% response after six months of treatment (25). We cannot entirely rule out that some of our patients may have responded at a later time point. However, many patients in our study were treated for more than three months with erenumab and we did not observe any significant changes of MHDs or AMDs in the last month of treatment with erenumab in these patients.

The lack of a control group does not allow us to rule out that the observed effects are the consequence of a placebo effect or are due to fluctuations of headache frequency during different cycles of migraine. However, the previous non-response to other preventives including one CGRP-R-mAb resulting in possibly lower expectations makes a placebo effect less likely (29). Subgroup analysis of the phase II study of erenumab in chronic migraine patients could demonstrate a lower placebo response in patients with prior treatment failure of at least one or two preventives (30). Patient records also revealed no change of lifestyle habits or the start of non-medical treatment that might have caused a reduction of headache frequency.

Another limitation is the evaluation of treatment response based on headache frequency and with no differentiated analysis of acute medication use. In particular, severely affected patients might also experience a benefit from treatment by achieving less severe attacks although headache frequency remains unchanged. This might be especially relevant for patients with daily headache. However, the non-standardized headache documentation in the present study did not allow the assessment of headache severity.

We cannot entirely exclude that headache frequency might also be affected by a treatment break of fewer than two half-lives (28 days) between the end of treatment with erenumab and the start of treatment with the CGRP-mAb in some patients. However, we assume that the effect of erenumab is limited in these patients as we only included non-responder to erenumab.

Our data support clinical observations and an increasingly common clinical practice of switching CGRP-mAb classes in non-responders to one class. However, our data is only generalizable to a part of the target population as we assessed only the switch from CGRP-R-mAb to a CGRP-mAb. If similar effects apply to the vice versa switch from a CGRP-mAb to a CGRP-R-mAb is a matter of future studies. Ideally, a prospective randomized controlled double-blind study should assess the CGRP-(R)-mAb switch to conduct a definite conclusion on this important question in daily migraine practice.

## Conclusion

Our retrospective findings in 25 treatment-resistant patients indicate that one out of three patients, who did not respond to the initial treatment with a CGRP-R-mAb, may benefit from the switch to a CGRP-mAb. Switching to a CGRP-mAb seems to be promising in particular in migraine patients who do not suffer from daily headache. Migraine patients with daily headache seem to be more likely to be treatment resistant.

## Clinical implications

- Our data suggest that switching CGRP-mAb classes in non-responders to a CGRP-R-mAb is a reasonable treatment strategy.
- A ≥30% response was achieved in one-third of the CGRP-R-mAb non-responders three months after a switch to a CGRP-mAb.
- None of the patients with daily headache showed a reduction of MHD after the switch from a CGRP-R-mAb to a CGRP-mAb.
